# Genome Sequence of *Klebsiella pneumoniae* YZUSK-4, a Bacterium Proposed as a Starter Culture for Fermented Meat Products

**DOI:** 10.1128/genomeA.00774-15

**Published:** 2015-07-23

**Authors:** Hai Yu, Yongqi Yin, Lin Xu, Ming Yan, Weiming Fang, Qingfeng Ge

**Affiliations:** a College of Food Science and Technology, Yangzhou University, Yangzhou, Jiangsu, China; b State Key Laboratory of Materials-Oriented Chemical Engineering, College of Biotechnology and Pharmaceutical Engineering, Nanjing University of Technology, Nanjing, Jiangsu, China

## Abstract

*Klebsiella pneumoniae* strain YZUSK-4, isolated from Chinese RuGao ham, is an efficient branched-chain aminotransferase-producing bacterium that can be used widely in fermented meat products to enhance flavor. The draft genome sequence of strain YZUSK-4 may provide useful genetic information on branched-chain amino acid aminotransferase production and branched-chain amino acid metabolism.

## GENOME ANNOUNCEMENT

Aroma is one of the most important characteristics for the overall quality of fermented meat products (1). Branched-chain aminotransferases (BcaT, EC 2.6.1.42) are responsible for the conversion of branched-chain amino acids (valine, leucine, and isoleucine) to their corresponding α-keto acids (2, 3), which are believed to be strongly linked to the formation of aroma compounds in fermented meats (4–6).

*Klebsiella pneumoniae* YZUSK-4, a natural strain without any modification, was isolated from the Chinese traditional RuGao ham. It can produce BcaT activity amounts as high as 42.69 ± 1.34 U/mL, and it can be widely used as a starter culture in the production of fermented meat products to enhance aroma and flavor.

The nucleotide sequence of the *Klebsiella pneumonia* strain YZUSK-4 genome was determined by using a HiSeq 2000 platform (Illumina, San Diego, CA, USA). A 300-bp paired-end library was constructed and sequenced, producing 8 million paired-end reads with read lengths of 100 bp.

A total of 162 contigs with a total size of 10,923,806 bp were assembled using Velvet version 1.2.07 (7), providing 56.66-fold coverage, ranging in size from 117 bp to 511,162 bp. The contigs were assembled into one circular contig by Geneious software version 8.1.4 (8) with the help of the reference genome of *Klebsiella pneumoniae* (GenBank accession number NC009648). The draft genome that resulted from this assembly is 5,081,069 bp long with a GC content of 54.28%.

Genome annotation was performed by the NCBI Prokaryotic Genome Annotation Pipeline (http://www.ncbi.nlm.nih.gov/genome/annotation_prok). A total of 4,751 genes, 4,587 coding sequences (CDSs), 88 pseudogenes, 1 rRNA, 66 tRNAs, and 5 noncoding RNAs (ncRNAs) were identified.

### Nucleotide sequence accession number.

The genome sequence of *Klebsiella pneumoniae* YZUSK-4 has been deposited in GenBank under the accession number CP011421.
